# Supplementary material for: A Comparative Study of Supervised Machine Learning Algorithms for the Prediction of Long-Range Chromatin Interactions
Source: Genes (Basel). 2020 Aug 24;11(9):985. doi: 10.3390/genes11090985 (PMC7563616; doi:10.3390/genes11090985)

Figure S1

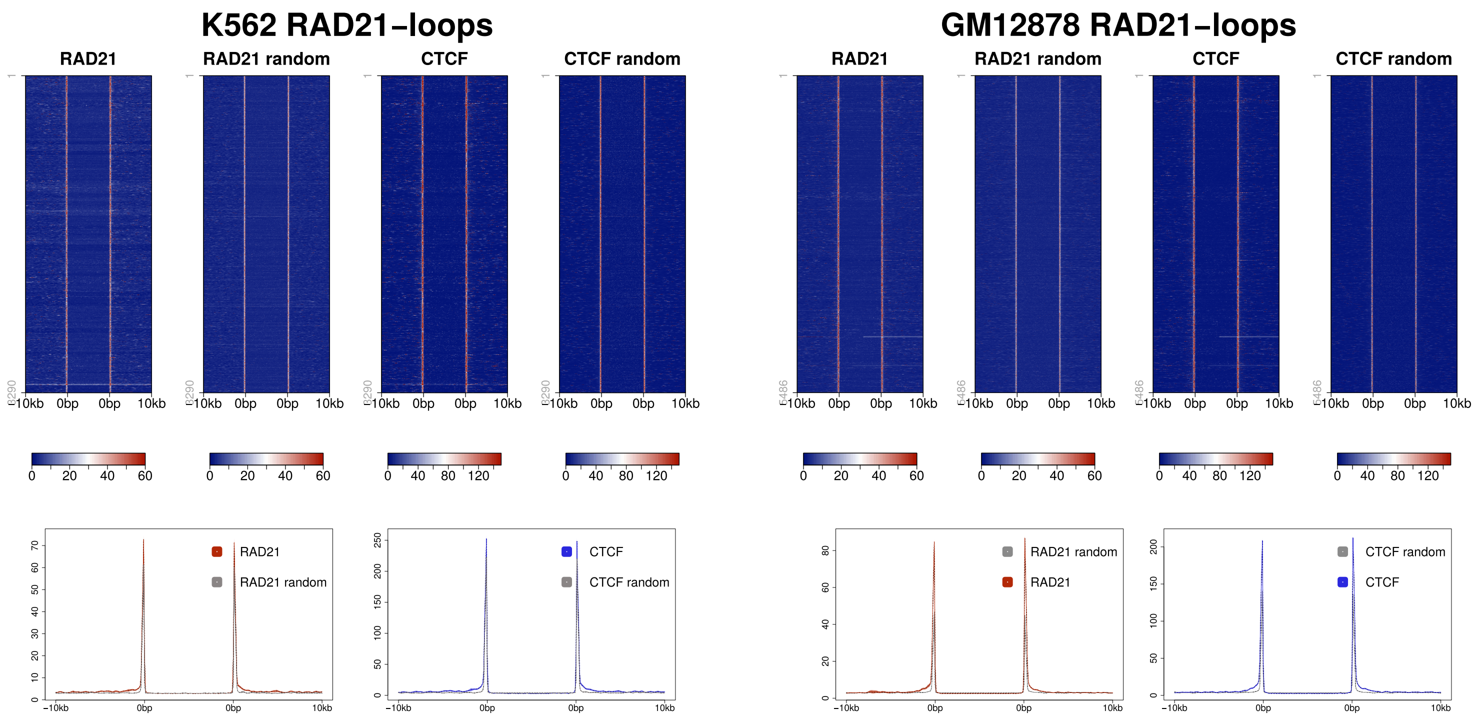

Figure S2

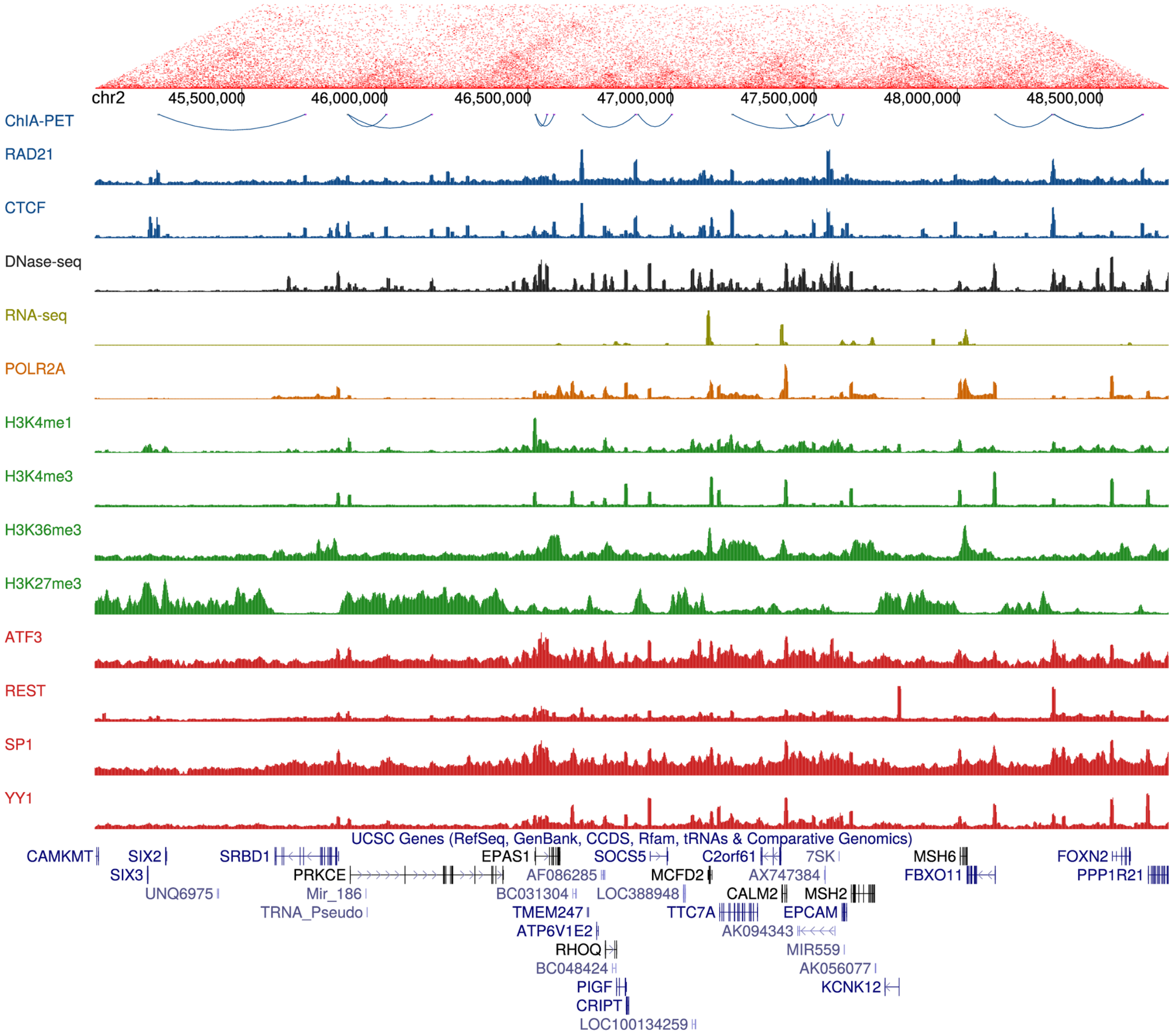

Figure S3

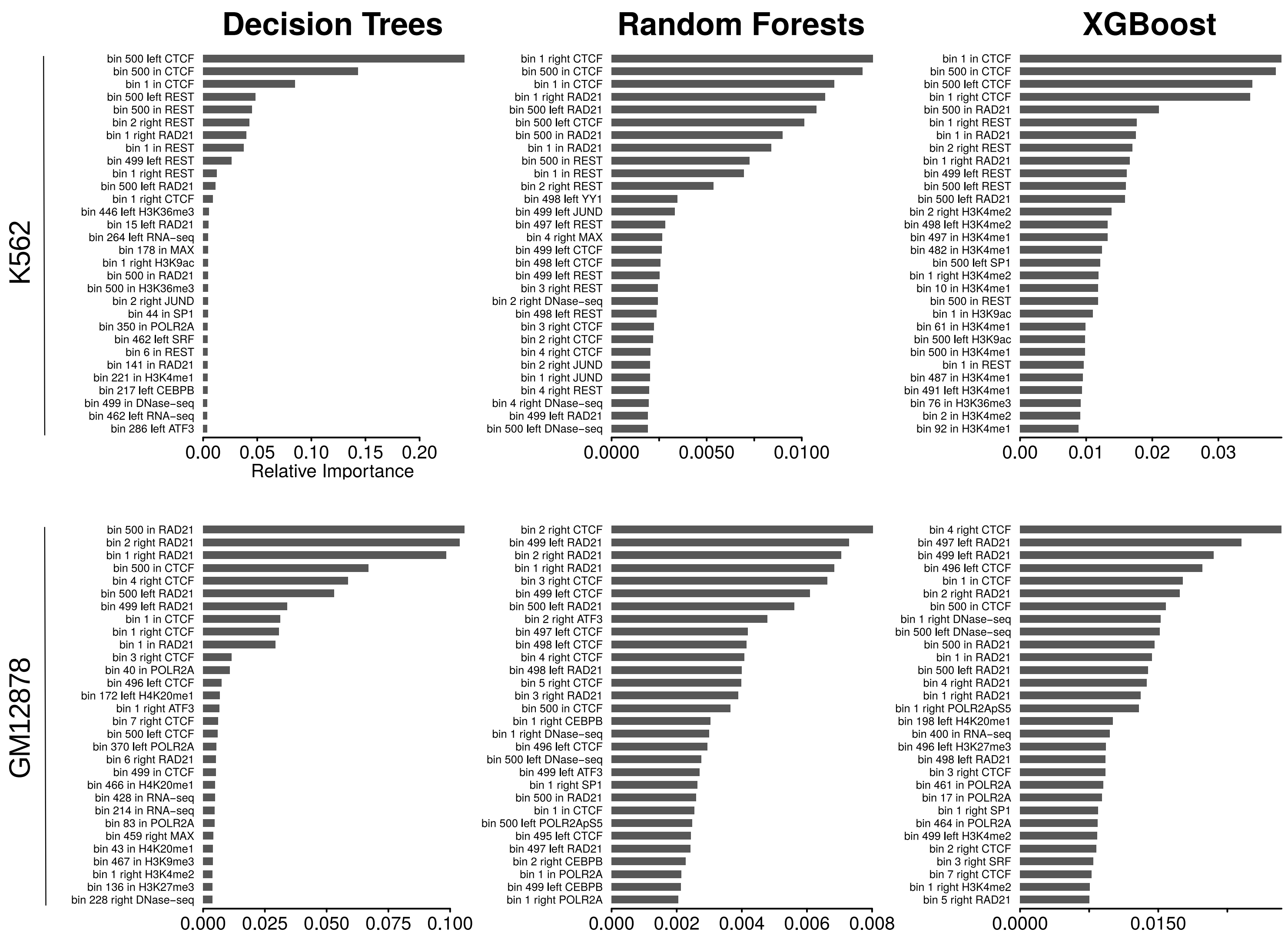

Decision Trees
Random Forests
XGBoost
K562
bin 500 left CTCF
bin 500 in CTCF
bin 1 in CTCF
bin 500 left REST
bin 500 in REST
bin 2 right REST
bin 1 right RAD21
bin 1 in REST
bin 499 left REST
bin 1 right REST
bin 500 left RAD21
bin 1 right CTCF
bin 446 left H3K36me3
bin 15 left RAD21
bin 264 left RNA−seq
bin 178 in MAX
bin 1 right H3K9ac
bin 500 in RAD21
bin 500 in H3K36me3
bin 2 right JUND
bin 44 in SP1
bin 350 in POLR2A
bin 462 left SRF
bin 6 in REST
bin 141 in RAD21
bin 221 in H3K4me1
bin 217 left CEBPB
bin 499 in DNase−seq
bin 462 left RNA−seq
bin 286 left ATF3
0.00
0.05
0.10
0.15
0.20
Relative Importance
bin 1 right CTCF
bin 500 in CTCF
bin 1 in CTCF
bin 1 right RAD21
bin 500 left RAD21
bin 500 left CTCF
bin 500 in RAD21
bin 1 in RAD21
bin 500 in REST
bin 1 in REST
bin 2 right REST
bin 498 left YY1
bin 499 left JUND
bin 497 left REST
bin 4 right MAX
bin 499 left CTCF
bin 498 left CTCF
bin 499 left REST
bin 3 right REST
bin 2 right DNase−seq
bin 498 left REST
bin 3 right CTCF
bin 2 right CTCF
bin 4 right CTCF
bin 2 right JUND
bin 1 right JUND
bin 4 right REST
bin 4 right DNase−seq
bin 499 left RAD21
bin 500 left DNase−seq
0.0000
0.0050
0.0100
bin 1 in CTCF
bin 500 in CTCF
bin 500 left CTCF
bin 1 right CTCF
bin 500 in RAD21
bin 1 right REST
bin 1 in RAD21
bin 2 right REST
bin 1 right RAD21
bin 499 left REST
bin 500 left REST
bin 500 left RAD21
bin 2 right H3K4me2
bin 498 left H3K4me2
bin 497 in H3K4me1
bin 482 in H3K4me1
bin 500 left SP1
bin 1 right H3K4me2
bin 10 in H3K4me1
bin 500 in REST
bin 1 in H3K9ac
bin 61 in H3K4me1
bin 500 left H3K9ac
bin 500 in H3K4me1
bin 1 in REST
bin 487 in H3K4me1
bin 491 left H3K4me1
bin 76 in H3K36me3
bin 2 in H3K4me2
bin 92 in H3K4me1
0.00
0.01
0.02
0.03
GM12878
bin 500 in RAD21
bin 2 right RAD21
bin 1 right RAD21
bin 500 in CTCF
bin 4 right CTCF
bin 500 left RAD21
bin 499 left RAD21
bin 1 in CTCF
bin 1 right CTCF
bin 1 in RAD21
bin 3 right CTCF
bin 40 in POLR2A
bin 496 left CTCF
bin 172 left H4K20me1
bin 1 right ATF3
bin 7 right CTCF
bin 500 left CTCF
bin 370 left POLR2A
bin 6 right RAD21
bin 499 in CTCF
bin 466 in H4K20me1
bin 428 in RNA−seq
bin 214 in RNA−seq
bin 83 in POLR2A
bin 459 right MAX
bin 43 in H4K20me1
bin 467 in H3K9me3
bin 1 right H3K4me2
bin 136 in H3K27me3
bin 228 right DNase−seq
0.000
0.025
0.050
0.075
0.100
bin 2 right CTCF
bin 499 left RAD21
bin 2 right RAD21
bin 1 right RAD21
bin 3 right CTCF
bin 499 left CTCF
bin 500 left RAD21
bin 2 right ATF3
bin 497 left CTCF
bin 498 left CTCF
bin 4 right CTCF
bin 498 left RAD21
bin 5 right CTCF
bin 3 right RAD21
bin 500 in CTCF
bin 1 right CEBPB
bin 1 right DNase−seq
bin 496 left CTCF
bin 500 left DNase−seq
bin 499 left ATF3
bin 1 right SP1
bin 500 in RAD21
bin 1 in CTCF
bin 500 left POLR2ApS5
bin 495 left CTCF
bin 497 left RAD21
bin 2 right CEBPB
bin 1 in POLR2A
bin 499 left CEBPB
bin 1 right POLR2A
0.000
0.002
0.004
0.006
0.008
bin 4 right CTCF
bin 497 left RAD21
bin 499 left RAD21
bin 496 left CTCF
bin 1 in CTCF
bin 2 right RAD21
bin 500 in CTCF
bin 1 right DNase−seq
bin 500 left DNase−seq
bin 500 in RAD21
bin 1 in RAD21
bin 500 left RAD21
bin 4 right RAD21
bin 1 right RAD21
bin 1 right POLR2ApS5
bin 198 left H4K20me1
bin 400 in RNA−seq
bin 496 left H3K27me3
bin 498 left RAD21
bin 3 right CTCF
bin 461 in POLR2A
bin 17 in POLR2A
bin 1 right SP1
bin 464 in POLR2A
bin 499 left H3K4me2
bin 2 right CTCF
bin 3 right SRF
bin 7 right CTCF
bin 1 right H3K4me2
bin 5 right RAD21
0.0000
0.0150

Figure S4

A) Decision Trees (K562)

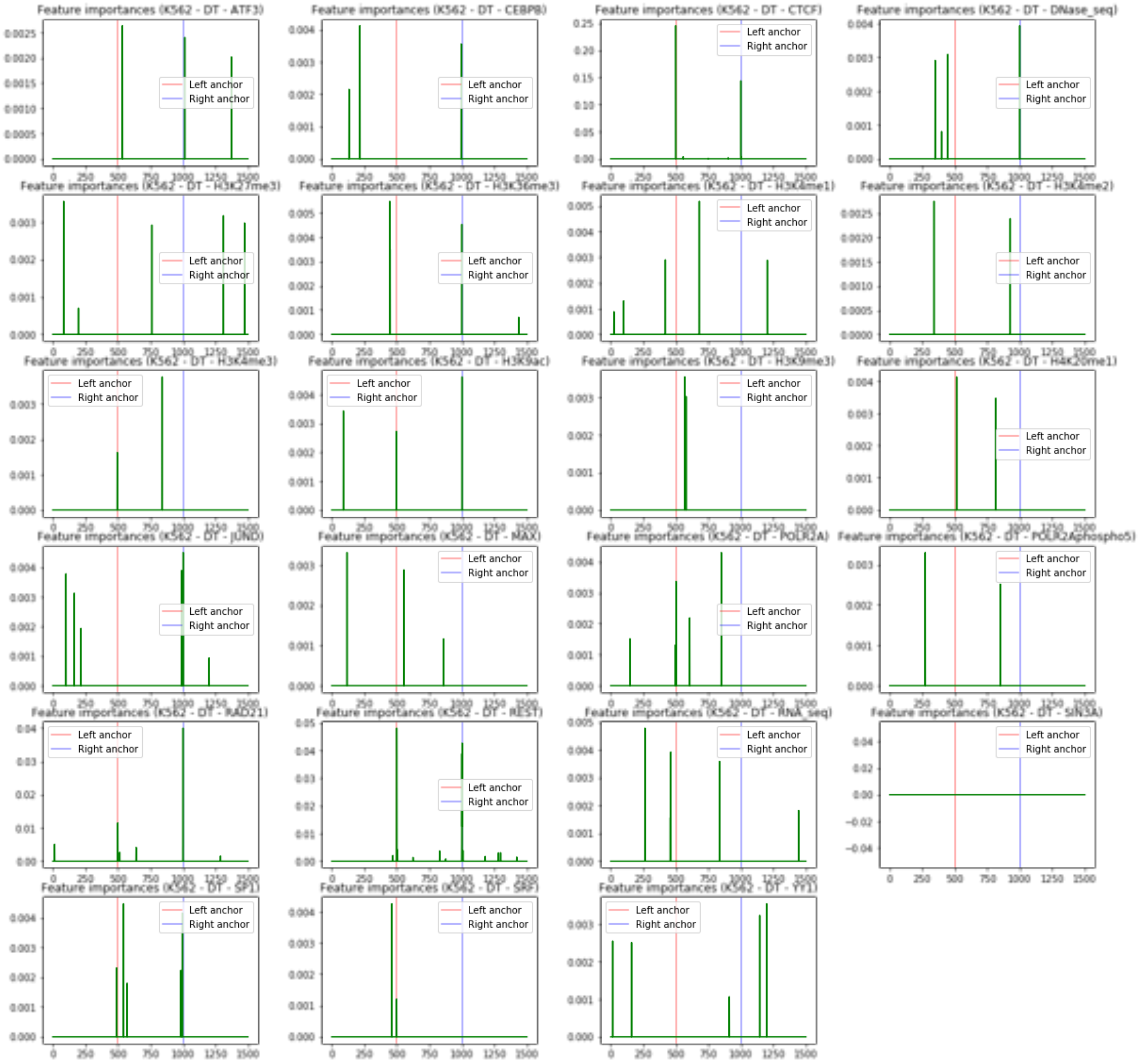

B) Random Forests (K562)

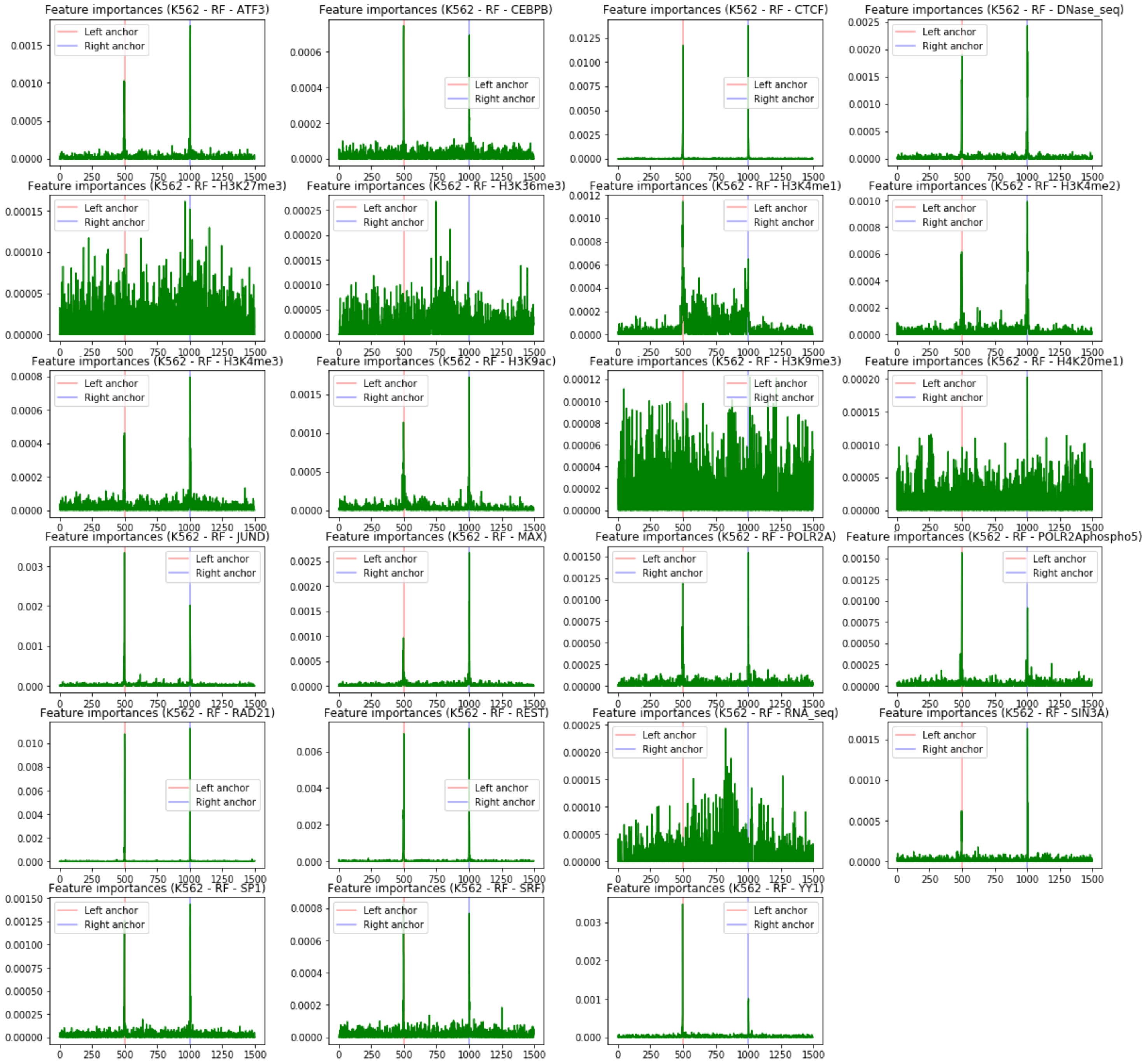

C) XGBoost (K562)

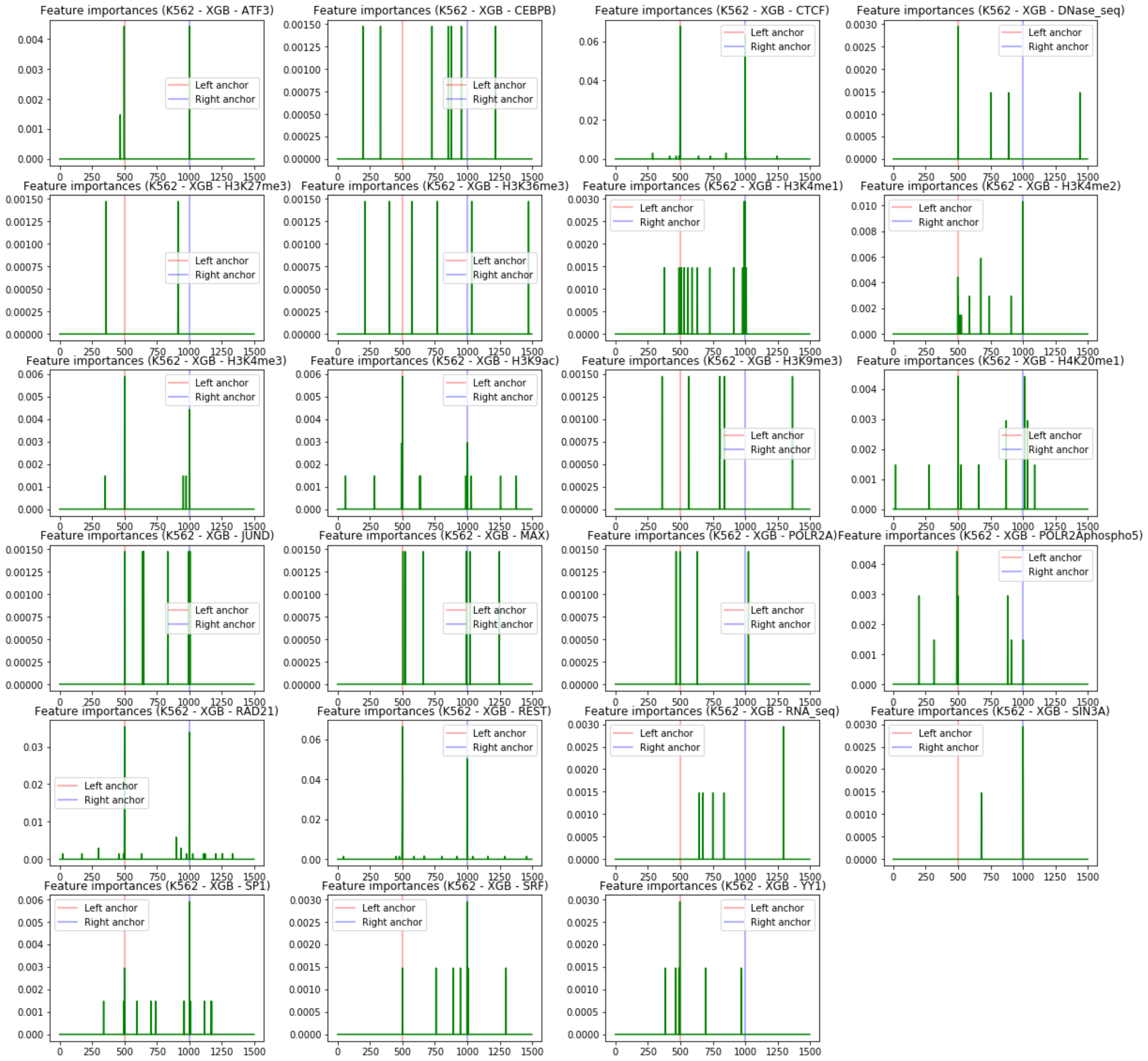

D) Decision Trees (GM12878)

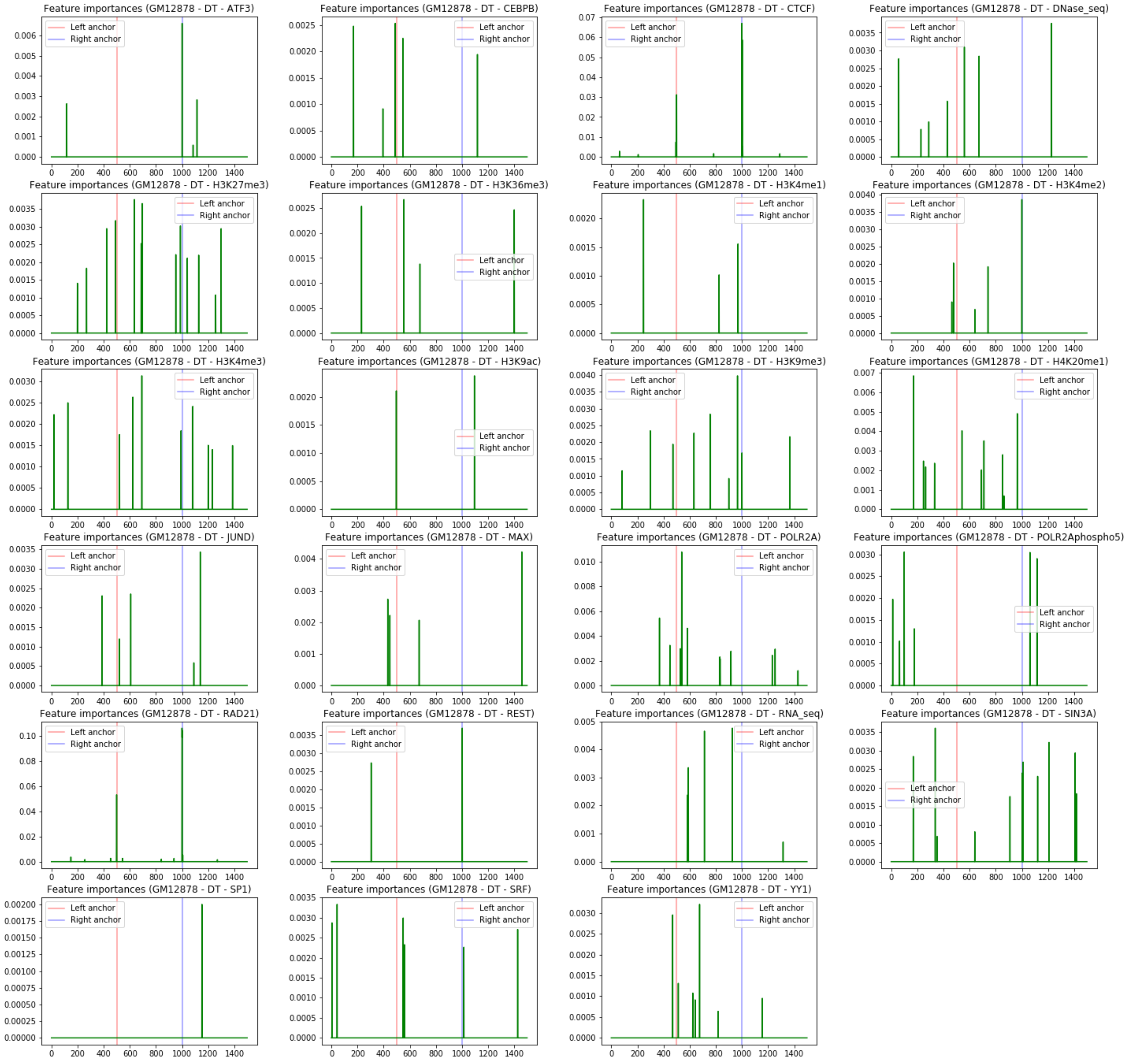

E) Random Forests (GM12878)

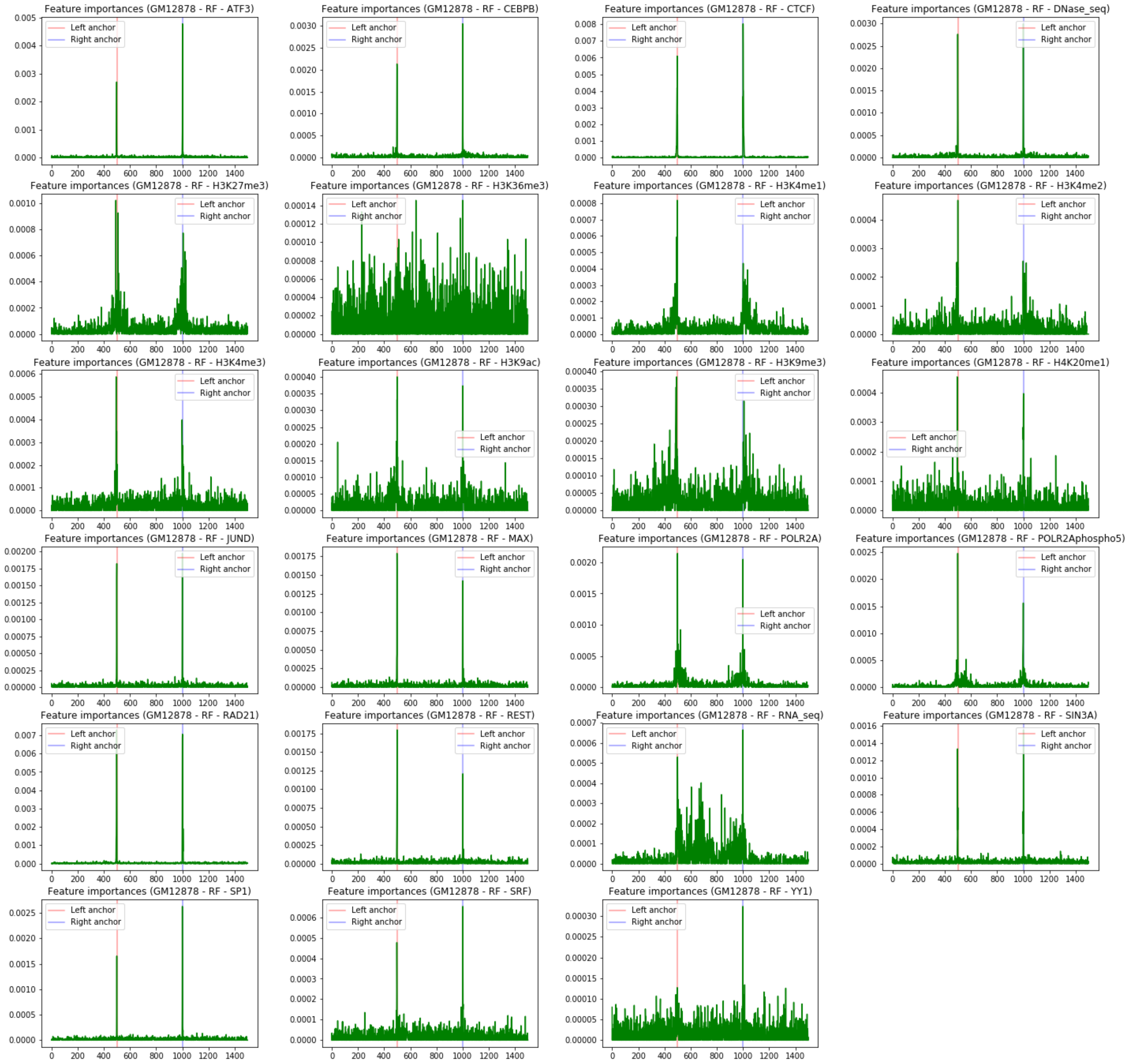

F) XGBoost (GM12878)

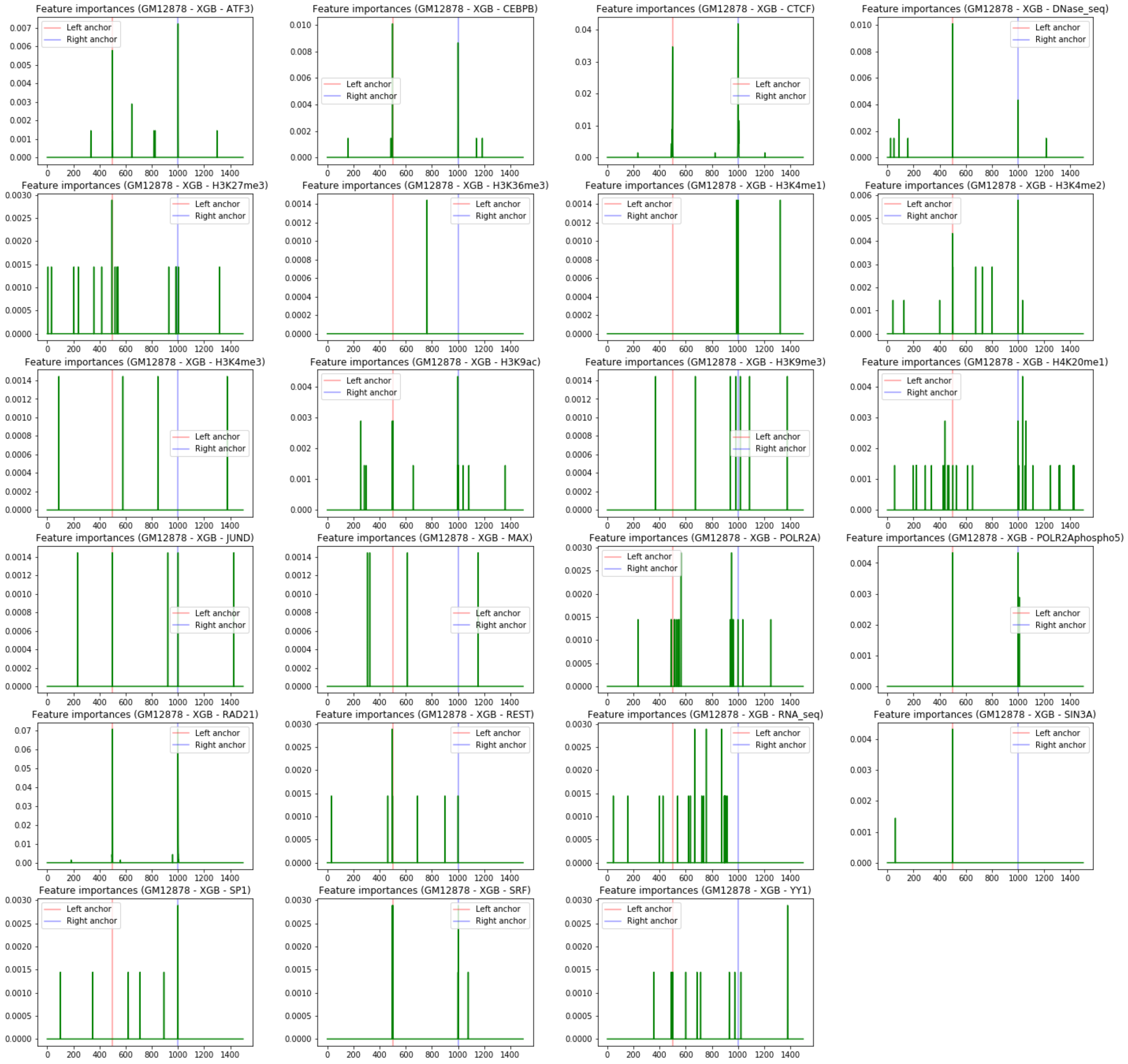

Figure S5

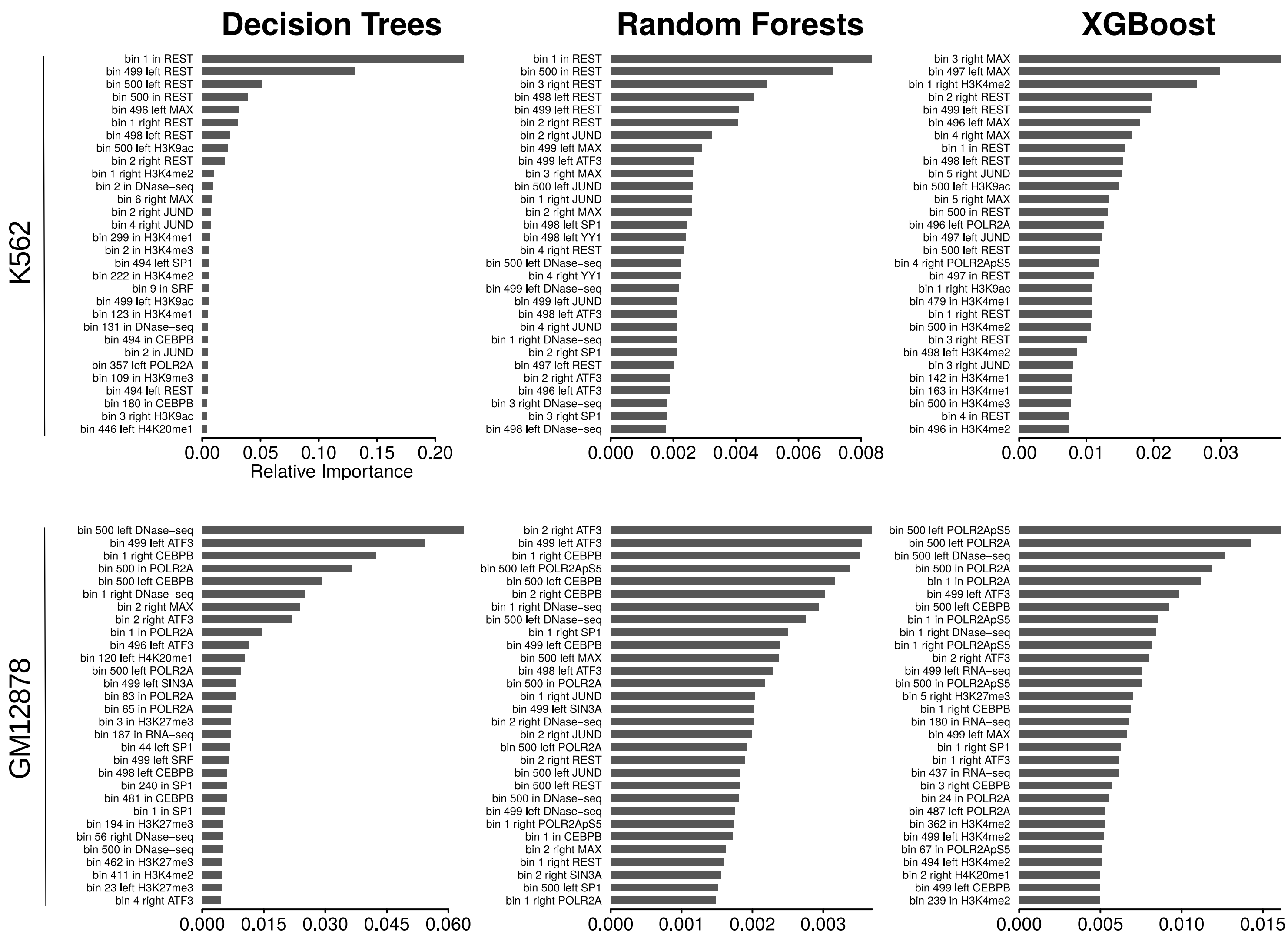

Decision Trees
Random Forests
XGBoost
K562
GM12878
Relative Importance
bin 1 in REST
bin 499 left REST
bin 500 left REST
bin 500 in REST
bin 496 left MAX
bin 1 right REST
bin 498 left REST
bin 500 left H3K9ac
bin 2 right REST
bin 1 right H3K4me2
bin 2 in DNase-seq
bin 6 right MAX
bin 2 right JUND
bin 4 right JUND
bin 299 in H3K4me1
bin 2 in H3K4me3
bin 494 left SP1
bin 222 in H3K4me2
bin 9 in SRF
bin 499 left H3K9ac
bin 123 in H3K4me1
bin 131 in DNase-seq
bin 494 in CEBPB
bin 2 in JUND
bin 357 left POLR2A
bin 109 in H3K9me3
bin 494 left REST
bin 180 in CEBPB
bin 3 right H3K9ac
bin 446 left H4K20me1
0.00 0.05 0.10 0.15 0.20
bin 1 in REST
bin 500 in REST
bin 3 right REST
bin 498 left REST
bin 499 left REST
bin 2 right REST
bin 2 right JUND
bin 499 left MAX
bin 499 left ATF3
bin 3 right MAX
bin 500 left JUND
bin 1 right JUND
bin 2 right MAX
bin 498 left SP1
bin 498 left YY1
bin 4 right REST
bin 500 left DNase-seq
bin 4 right YY1
bin 499 left DNase-seq
bin 499 left JUND
bin 498 left ATF3
bin 4 right JUND
bin 1 right DNase-seq
bin 2 right SP1
bin 497 left REST
bin 2 right ATF3
bin 496 left ATF3
bin 3 right DNase-seq
bin 3 right SP1
bin 498 left DNase-seq
0.000 0.002 0.004 0.006 0.008
bin 3 right MAX
bin 497 left MAX
bin 1 right H3K4me2
bin 2 right REST
bin 499 left REST
bin 496 left MAX
bin 4 right MAX
bin 1 in REST
bin 498 left REST
bin 5 right JUND
bin 500 left H3K9ac
bin 5 right MAX
bin 500 in REST
bin 496 left POLR2A
bin 497 left JUND
bin 500 left REST
bin 4 right POLR2ApS5
bin 497 in REST
bin 1 right H3K9ac
bin 479 in H3K4me1
bin 1 right REST
bin 500 in H3K4me2
bin 3 right REST
bin 498 left H3K4me2
bin 3 right JUND
bin 142 in H3K4me1
bin 163 in H3K4me1
bin 500 in H3K4me3
bin 4 in REST
bin 496 in H3K4me2
0.00 0.01 0.02 0.03
bin 500 left DNase-seq
bin 499 left ATF3
bin 1 right CEBPB
bin 500 in POLR2A
bin 500 left CEBPB
bin 1 right DNase-seq
bin 2 right MAX
bin 2 right ATF3
bin 1 in POLR2A
bin 496 left ATF3
bin 120 left H4K20me1
bin 500 left POLR2A
bin 499 left SIN3A
bin 83 in POLR2A
bin 65 in POLR2A
bin 3 in H3K27me3
bin 187 in RNA-seq
bin 44 left SP1
bin 499 left SRF
bin 498 left CEBPB
bin 240 in SP1
bin 481 in CEBPB
bin 1 in SP1
bin 194 in H3K27me3
bin 56 right DNase-seq
bin 500 in DNase-seq
bin 462 in H3K27me3
bin 411 in H3K4me2
bin 23 left H3K27me3
bin 4 right ATF3
0.000 0.015 0.030 0.045 0.060
bin 2 right ATF3
bin 499 left ATF3
bin 1 right CEBPB
bin 500 left POLR2ApS5
bin 500 left CEBPB
bin 2 right CEBPB
bin 1 right DNase-seq
bin 500 left DNase-seq
bin 1 right SP1
bin 499 left CEBPB
bin 500 left MAX
bin 498 left ATF3
bin 500 in POLR2A
bin 1 right JUND
bin 499 left SIN3A
bin 2 right DNase-seq
bin 2 right JUND
bin 500 left POLR2A
bin 2 right REST
bin 500 left JUND
bin 500 left REST
bin 500 in DNase-seq
bin 499 left DNase-seq
bin 1 right POLR2ApS5
bin 1 in CEBPB
bin 2 right MAX
bin 1 right REST
bin 2 right SIN3A
bin 500 left SP1
bin 1 right POLR2A
0.000 0.001 0.002 0.003
bin 500 left POLR2ApS5
bin 500 left POLR2A
bin 500 left DNase-seq
bin 500 in POLR2A
bin 1 in POLR2A
bin 499 left ATF3
bin 500 left CEBPB
bin 1 in POLR2ApS5
bin 1 right DNase-seq
bin 1 right POLR2ApS5
bin 2 right ATF3
bin 499 left RNA-seq
bin 500 in POLR2ApS5
bin 5 right H3K27me3
bin 1 right CEBPB
bin 180 in RNA-seq
bin 499 left MAX
bin 1 right SP1
bin 1 right ATF3
bin 437 in RNA-seq
bin 3 right CEBPB
bin 24 in POLR2A
bin 487 left POLR2A
bin 362 in H3K4me2
bin 499 left H3K4me2
bin 67 in POLR2ApS5
bin 494 left H3K4me2
bin 2 right H4K20me1
bin 499 left CEBPB
bin 239 in H3K4me2
0.000 0.005 0.010 0.015

Figure S6

A) Decision Trees (K562)

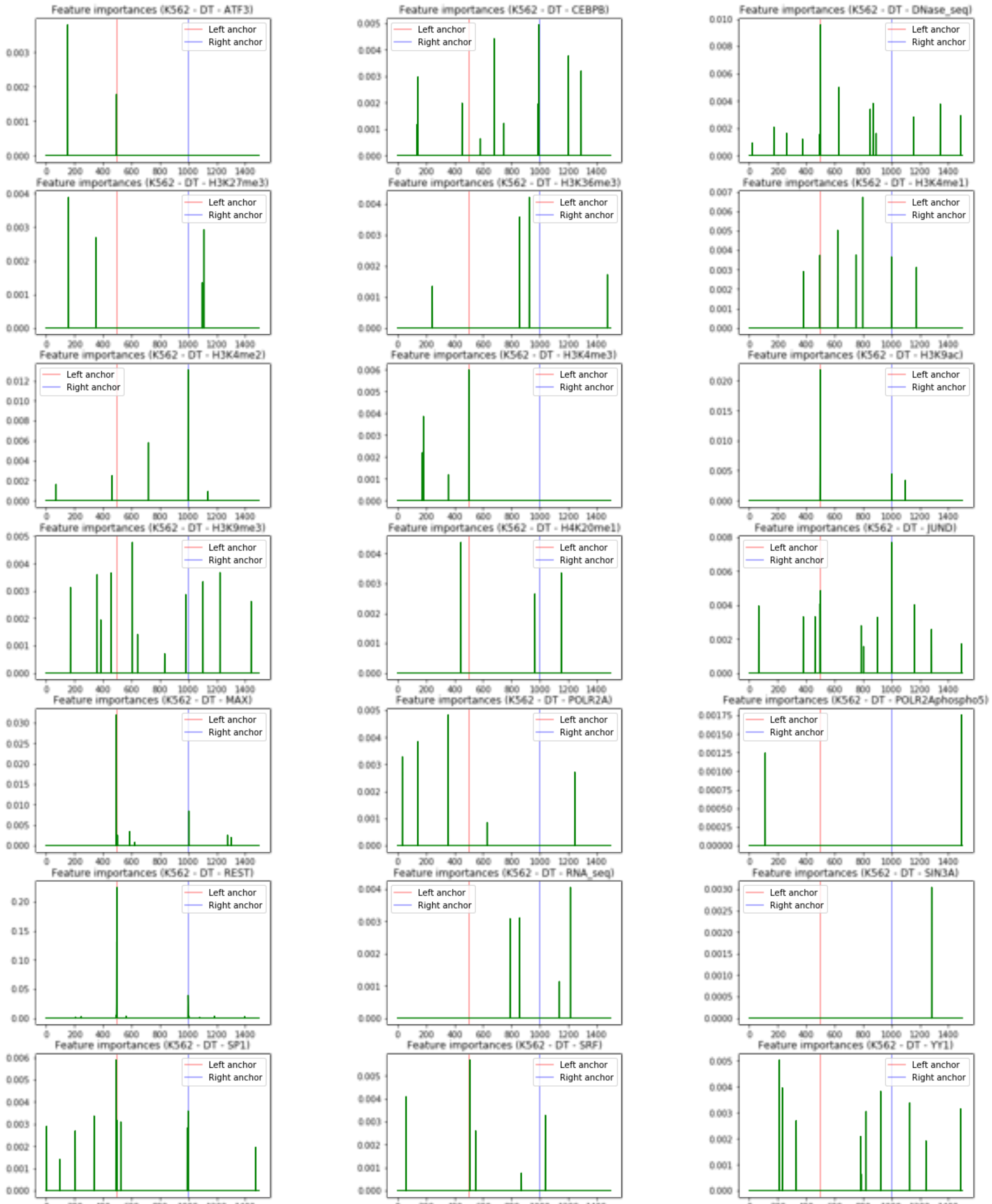

B) Random Forests (K562)

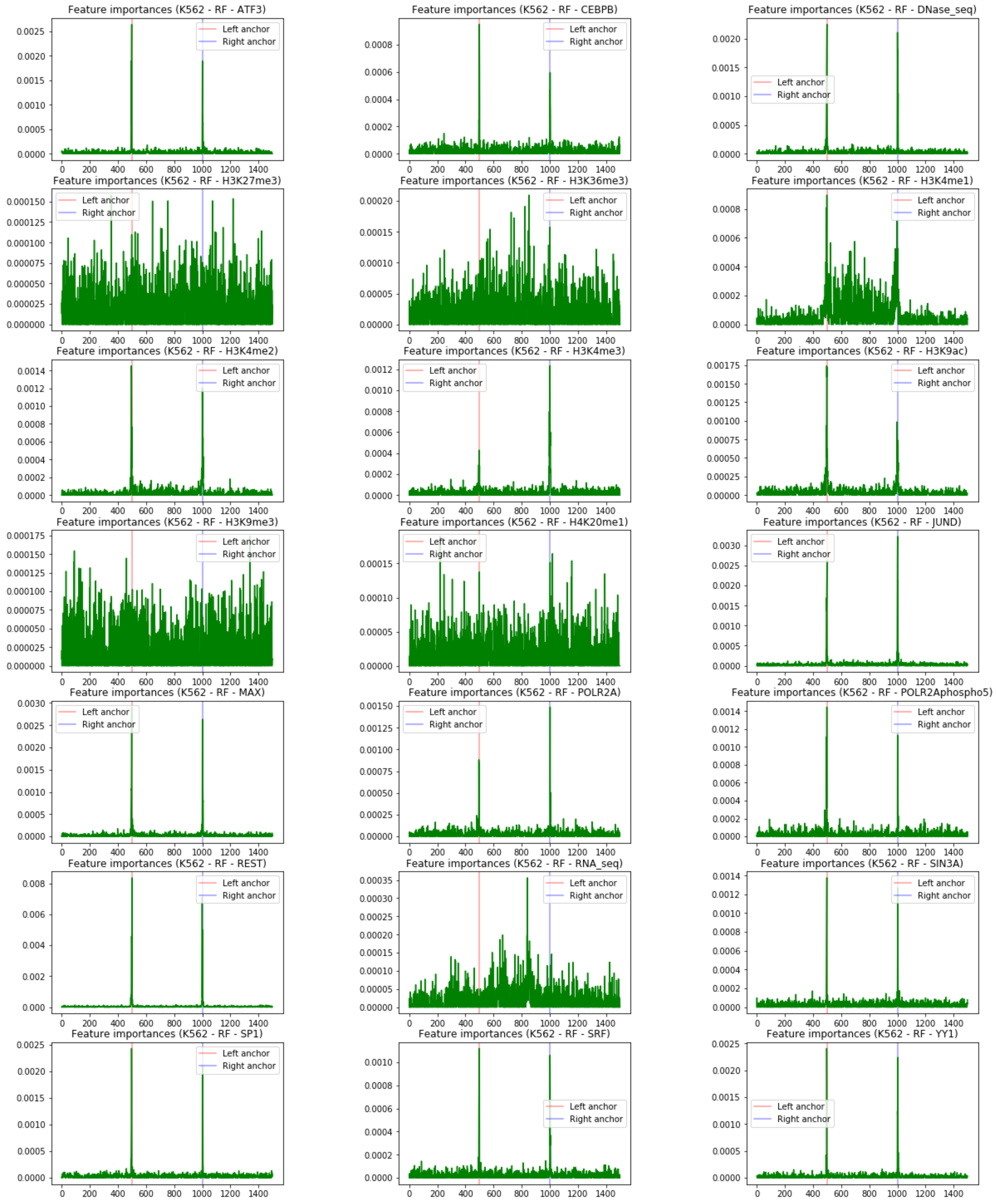

C) XGBoost (K562)

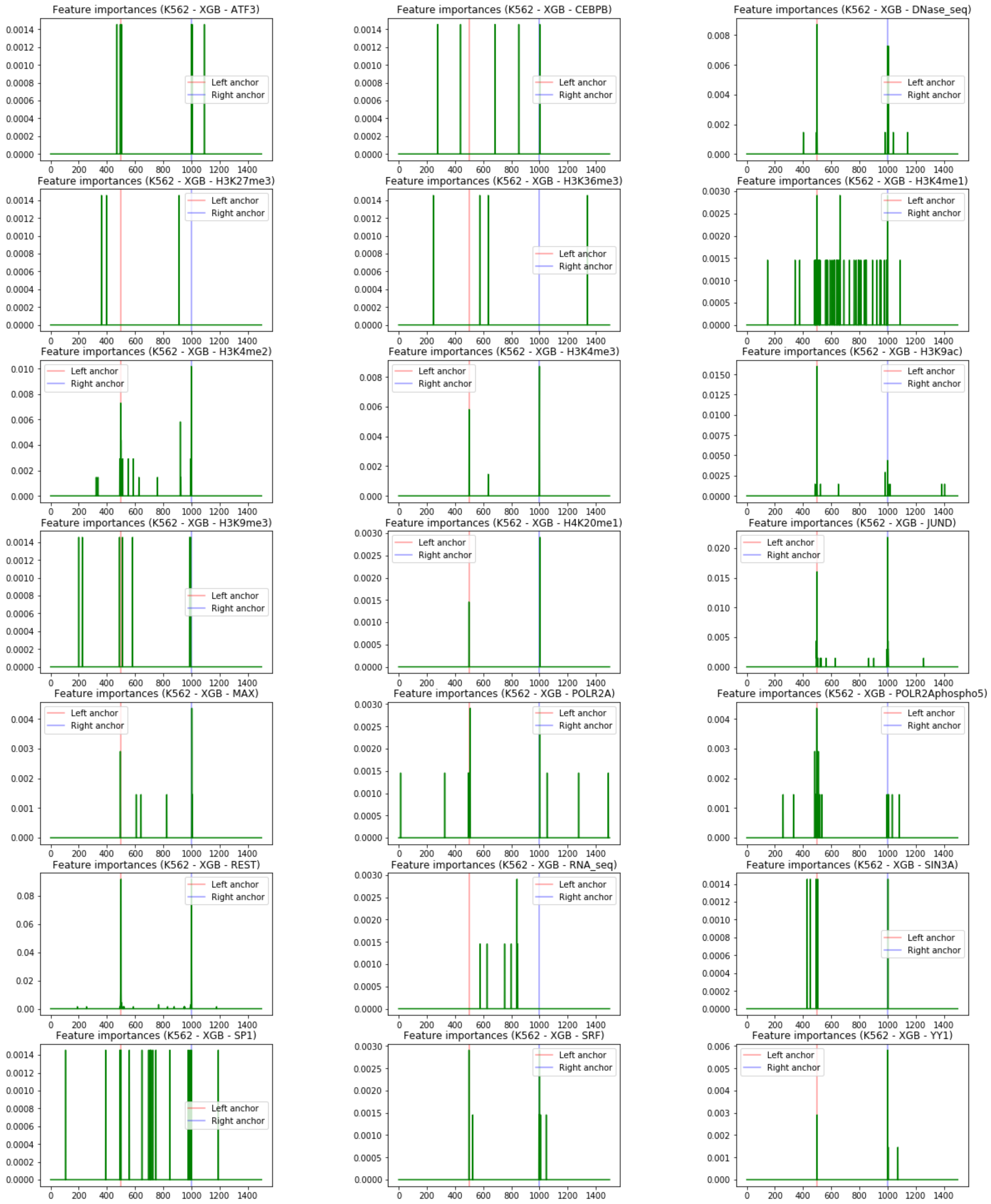

D) Decision Trees (GM12878)

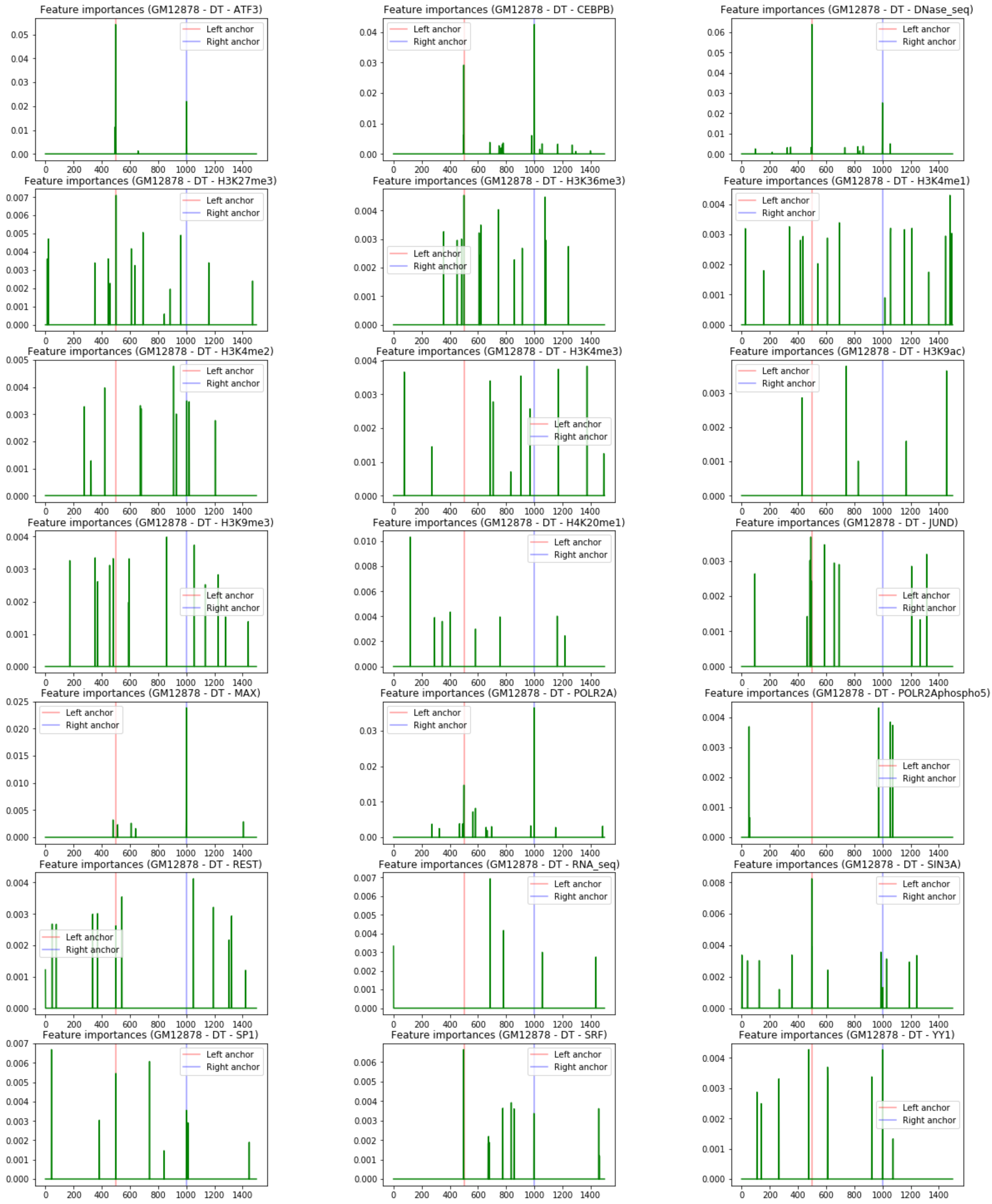

E) Random Forests (GM12878)

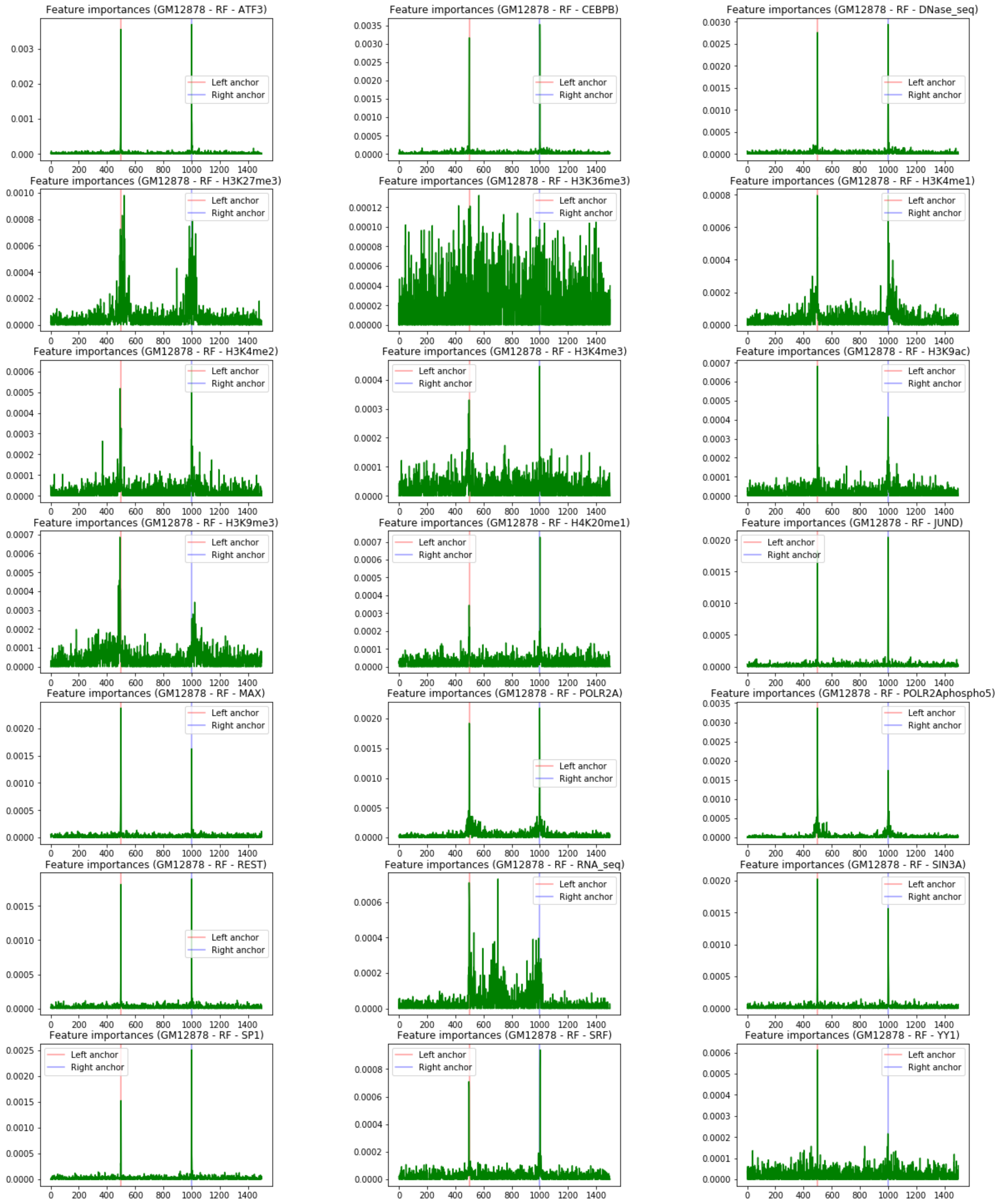

F) XGBoost (GM12878)

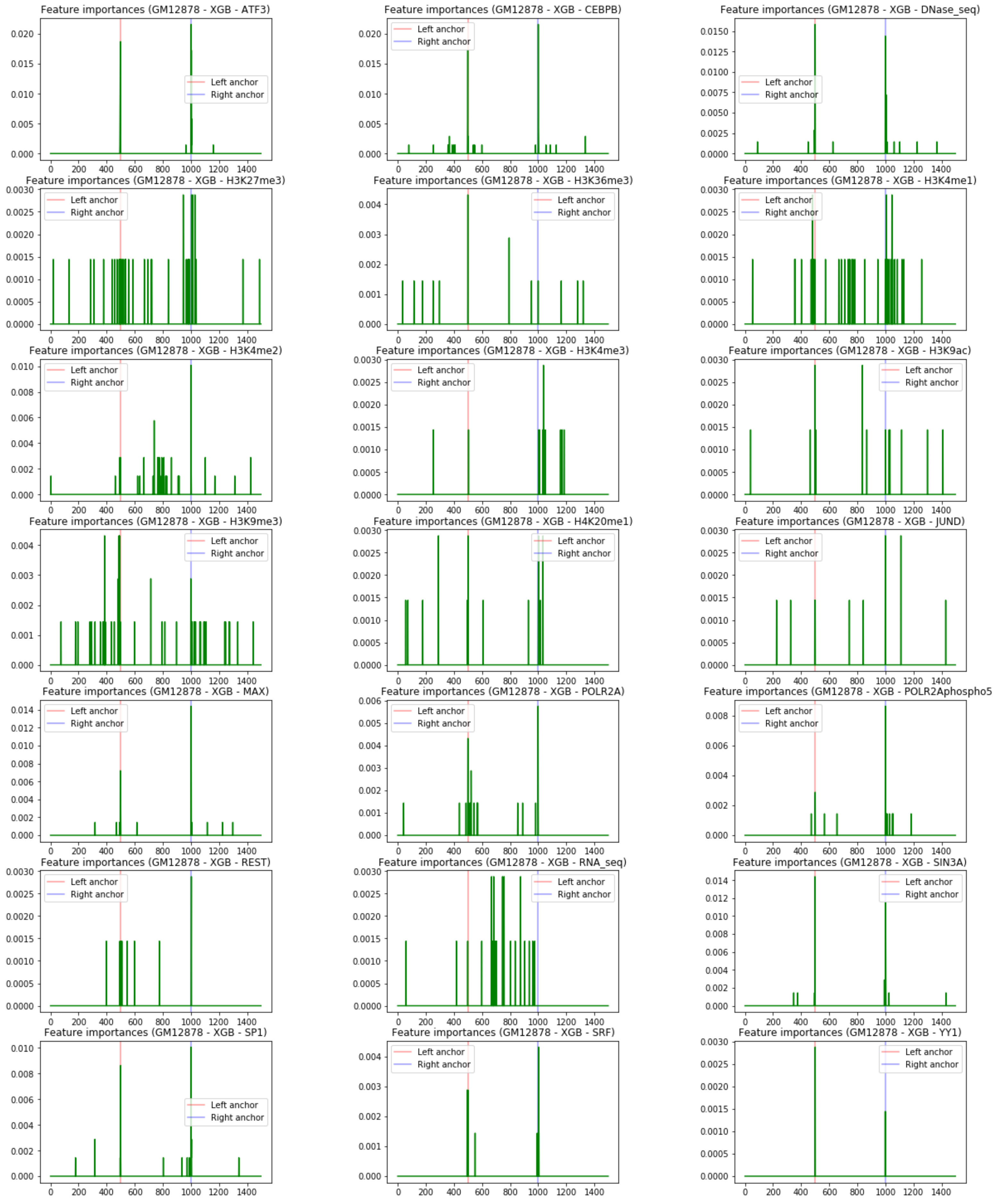

Figure S7

A) Architectural

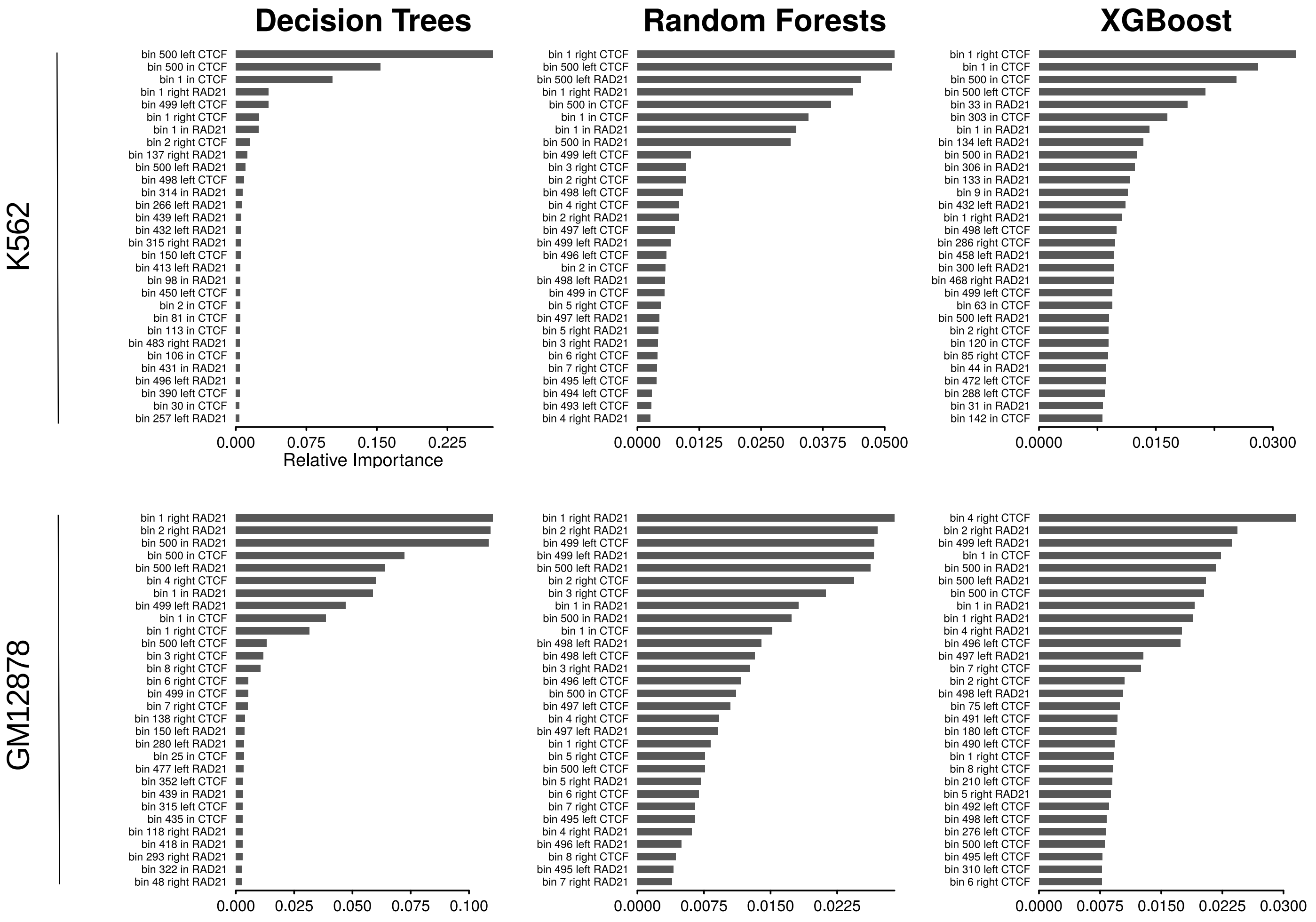

B) Transcription factors

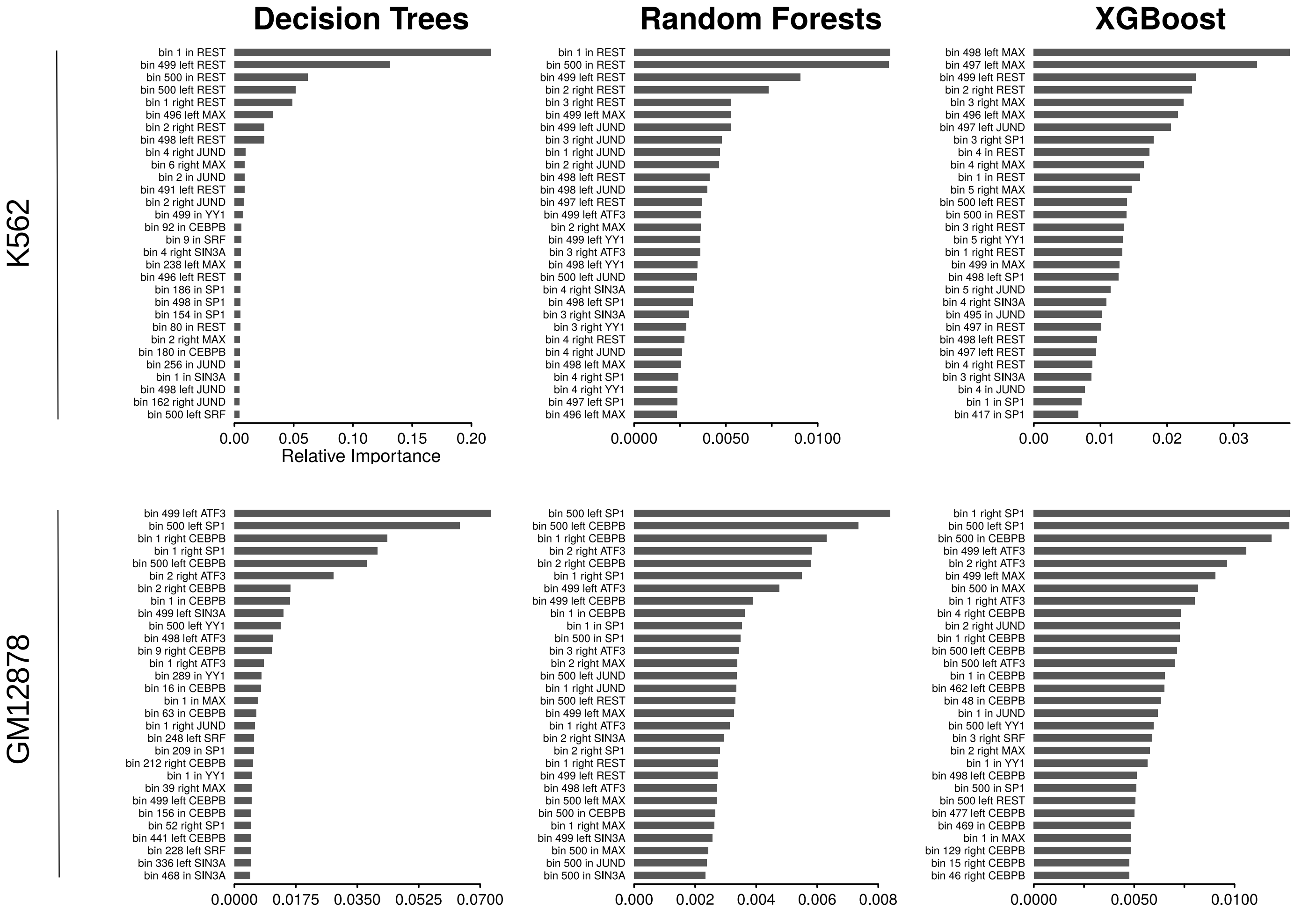

C) Architectural-anchors

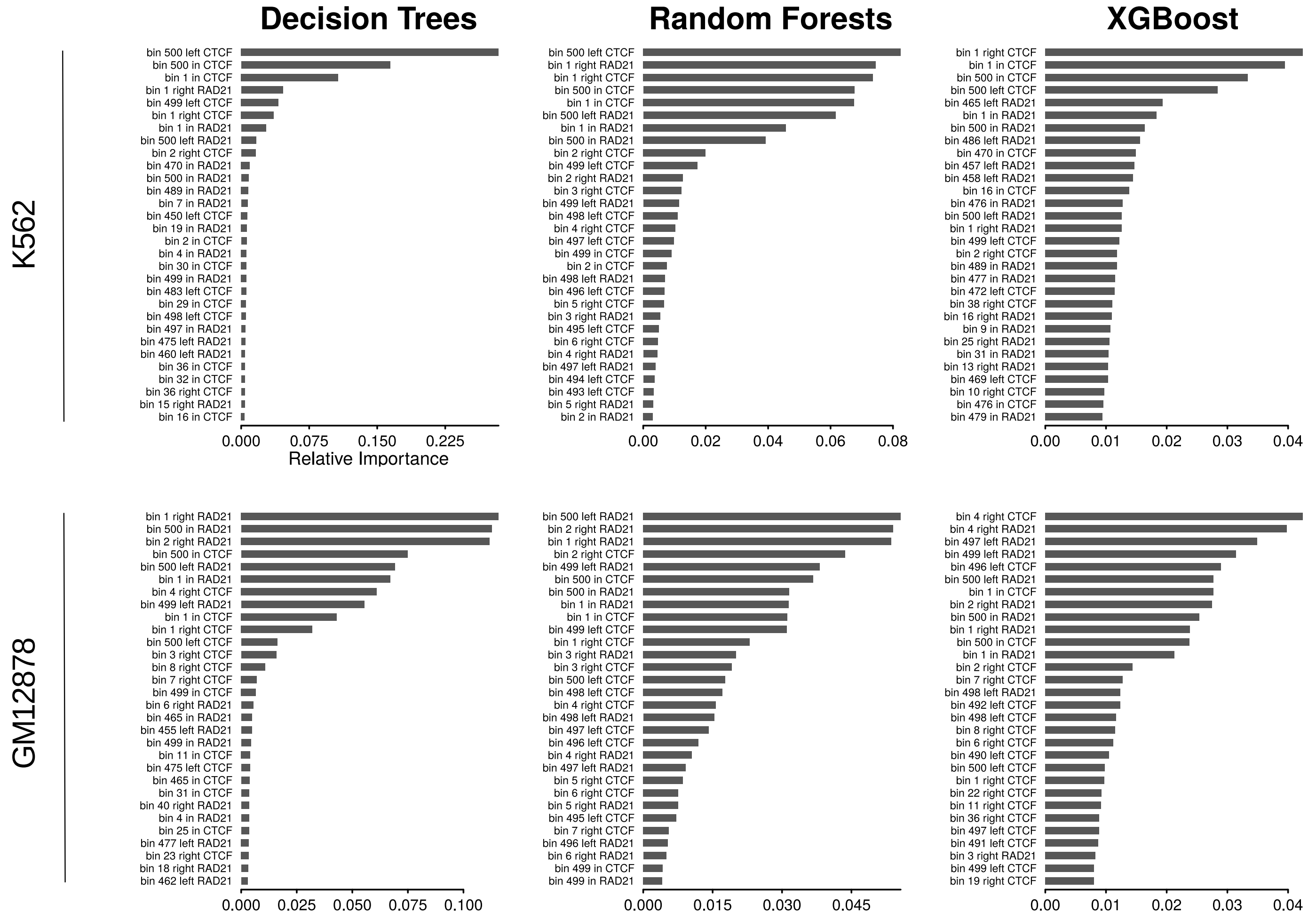

D) Transcription factors-anchors

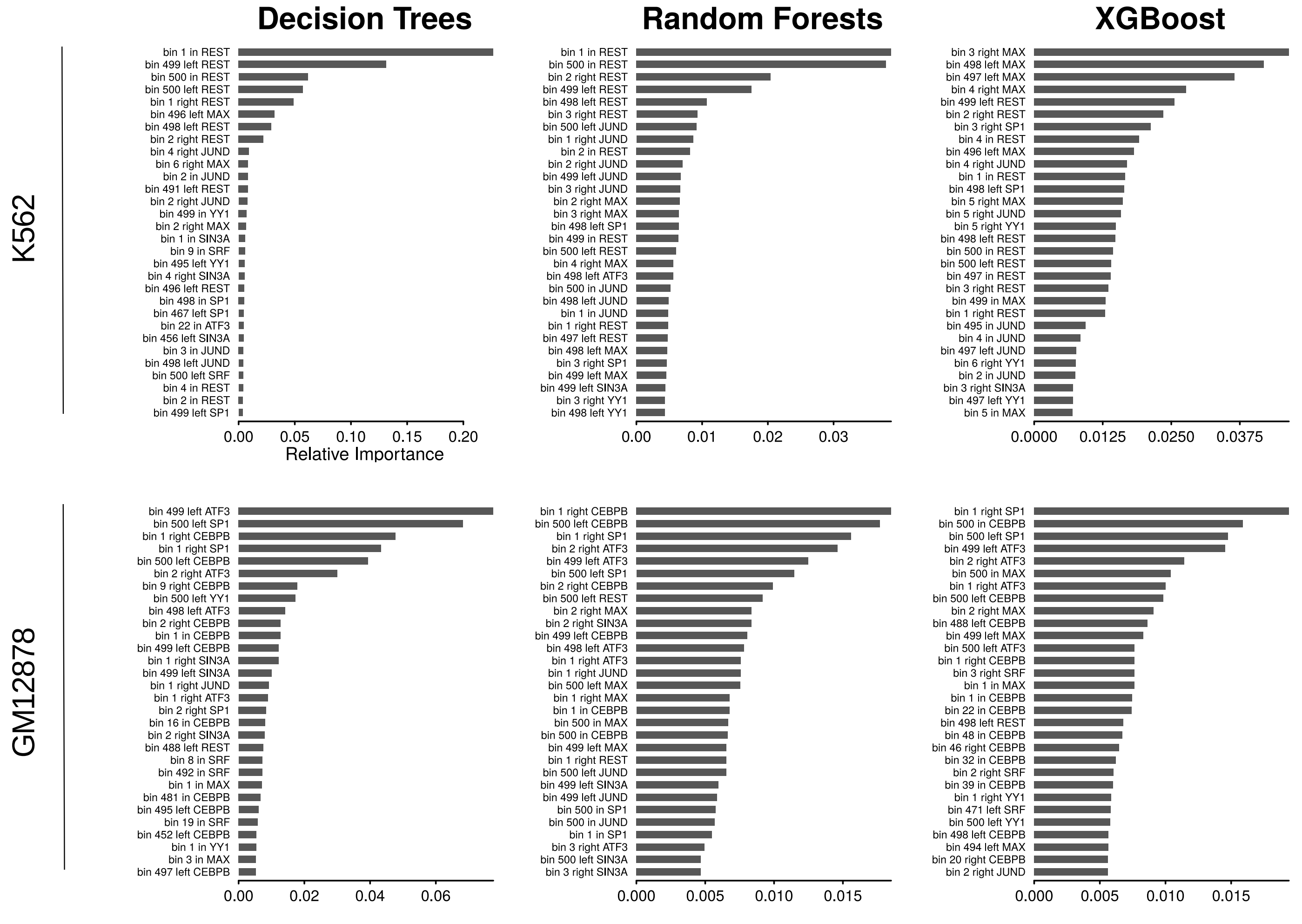

Supplement: Supplementary file 1 [file genes-11-00985-s001.pdf]
